# Inhibition of autophagy; an opportunity for the treatment of cancer resistance

**DOI:** 10.3389/fcell.2023.1177440

**Published:** 2023-06-09

**Authors:** Asha Tonkin-Reeves, Charlett M. Giuliani, John T. Price

**Affiliations:** ^1^ Faculty of Medicine, Dentistry and Health Sciences, University of Melbourne, Melbourne, VIC, Australia; ^2^ Institute for Health and Sport, Victoria University, Melbourne, VIC, Australia; ^3^ Australian Institute for Musculoskeletal Science (AIMSS), Victoria University and Western Health, Melbourne, VIC, Australia; ^4^ Monash Biomedicine Discovery Institute and Department of Biochemistry and Molecular Biology, Monash University, Melbourne, VIC, Australia

**Keywords:** autophagy, drug resisitance, therapeutic resistance, cancer, autophagy mechanisms

## Abstract

The process of macroautophagy plays a pivotal role in the degradation of long-lived, superfluous, and damaged proteins and organelles, which are later recycled for cellular use. Normal cells rely on autophagy to combat various stressors and insults to ensure survival. However, autophagy is often upregulated in cancer cells, promoting a more aggressive phenotype that allows mutated cells to evade death after exposure to therapeutic treatments. As a result, autophagy has emerged as a significant factor in therapeutic resistance across many cancer types, with underlying mechanisms such as DNA damage, cell cycle arrest, and immune evasion. This review provides a comprehensive summary of the role of autophagy in therapeutic resistance and the limitations of available autophagic inhibitors in cancer treatment. It also highlights the urgent need to explore new inhibitors that can synergize with existing therapies to achieve better patient treatment outcomes. Advancing research in this field is crucial for developing more effective treatments that can help improve the lives of cancer patients.

## Introduction

Cancer remains one of the most significant health concerns and a leading cause of death, with a reported worldwide mortality rate of nearly 10 million in 2020 (World Health Organisation, 2020). Advances in early detection and treatments have increased overall survival, with most improvements seen in low-grade cancers. Despite this, survival rates in metastatic cancers remain relatively poor, which can be attributed to the complex nature of treating cancers and therapeutic resistance (Weiss et al., 2022).

Therapeutic resistance is the resistance towards a therapy that the cells either innately have or develop after exposure to the treatment. Many cellular adaptions may affect this, one of which is autophagy, the focus of this review. Addressing the role of autophagy in drug resistance has been a primary focus of efforts in recent years. Autophagy is a molecular process where organelles and non-essential proteins are degraded to provide energy and nutrients for the cell in response to cellular and environmental stresses. There are three mammalian pathways: chaperone-mediated autophagy (CMA), microautophagy and macroautophagy. Of these three types of autophagy, macroautophagy has been the most studied and will be referred to as autophagy hereafter. All three pathways vary in how they transport and target proteins, but they all culminate and achieve the degradation of materials at the lysosome.

Understanding the role of autophagy in various cancers has led to the research and development of autophagy and lysosomal inhibitors, where pre-clinical studies have demonstrated promising results, with many agents progressing to clinical trials as stand-alone treatments or in combination with standard of care therapeutics. Of note, most current lysosomal inhibitors are repurposed agents, previously being used for diseases like malaria, and have thus progressed to the clinic faster than those in early development. The research discussed in this review highlights the advances within this area as well as the potential limitations of their use. Examination of recent evidence for their use will be used to reveal current knowledge gaps that contribute to their current limitations and how these can be potentially overcome. Thereby, gaining a better understanding and sense of the autophagic process being an effective treatment target for advanced cancers in the future.

### Macroautophagy

Autophagy is one of two central and essential degradative cellular processes, the other being the highly specialised and specific proteasomal degradation pathway (ubiquitin-proteasome system). Autophagy is mainly responsible for degrading superfluous, long-lived, damaged, dysfunctional proteins, protein aggregates, oligomers, and organelles. This process helps to maintain cellular homeostasis and is essential in preventing the toxic build-up of these materials.

It primarily functions to break down proteins and organelles into their base components, like glucose, ATP, amino acids and fatty acids (Lin et al., 2021), as a source of recyclable molecules for use by the cell. Creating these substrates helps support the cell’s metabolism and also helps supply the building blocks for the synthesis of new materials (Kaushik et al., 2010). In addition, autophagy has several other cytoprotective roles that include but are not limited to removing toxic proteins, damaging DNA, eliminating invasive microbes, and participating in antigen presentation. Its activation is stimulated by the traditional starvation stimuli and several additional stresses, including growth factor deprivation, hypoxia, reactive oxygen species (ROS), DNA damage and intracellular pathogens (He and Klionsky, 2009; Ravikumar et al., 2009; Ma et al., 2013; Vessoni et al., 2013; Yonekawa and Thorburn, 2013). These roles make macroautophagy a unique cell survival mechanism that prevents oncogenic/tumorigenic transformations at the early stages of cancer development. However, it becomes fundamental at later stages of cancer progression as it maintains an aggressive cancer phenotype through providing an alternate nutrient and amino acid source. Furthermore, autophagy has been shown to support the cellular changes observed in the aggressive phenotypes such as the epithelial to mesenchymal transition (Fung et al., 2008). This “pathologic” autophagic activity has been shown to reduce the efficacy of cancer treatments facilitating resistance by various mechanisms, as outlined in this review.

## Mechanisms

**FIGURE 1:**
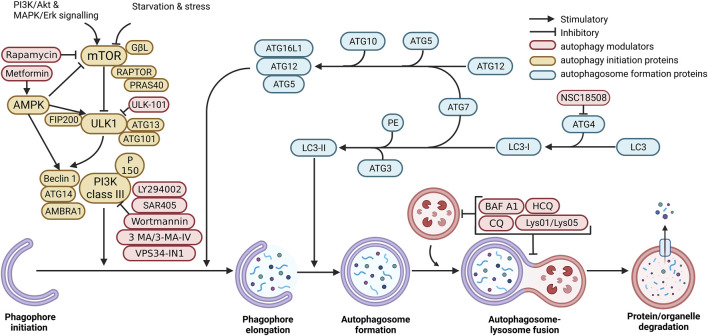
Mammalian macroautophagy and autophagy modulators. Autophagosome formation occurs when mTOR and other signalling pathways activate the ULK1 complex. Subsequently, the phagophore is elongated by stimulating the Atg5-Atg12-Atg16 complex. This process continues onto the maturation of the autophagosome with the ubiquitin-like reaction, which converts LC3-I to LC3-II. After this, the mature autophagosome fuses with the lysosome to form the autolysosome, which utilises enzymes to degrade proteins and organelles encapsulated by the autophagosome. The resulting material is recycled. Created with BioRender.com.

Macroautophagy is a process where an autophagosome engulfs organelles and proteins by developing a double membrane vesicle called the phagophore, which delivers cytoplasmic materials to the lysosome (illustrated in Figure 1) (Itakura and Mizushima, 2010). The lysosome and the autophagosome fuse to form the autolysosome, making it possible to degrade and recycle the cytoplasmic proteins and organelles (Itakura and Mizushima, 2010). The autophagosome is regulated by stress stimuli, which initiates a stepwise process: initiation/nucleation, elongation, and maturation of the phagophore (the precursor to the autophagosome) (Ravikumar et al., 2009). The process detailed below is known as the canonical pathway, as opposed to the non-canonical pathway that will not be discussed in this review and is a process that achieves degradation *via* the lysosome without the activation of hierarchical Atg’s.

### Phagophore initiation/nucleation

The phagophore membrane formation is still poorly understood in mammals. The formation of the autophagosome from the phagophore is initiated at multiple sites throughout the cytoplasm (Itakura and Mizushima, 2010; Feng et al., 2013; Puri et al., 2013). The membrane components of the phagophore have been suggested to be derived from numerous sources (Nakatogawa, 2020), including the endoplasmic reticulum–Golgi intermediate compartment (ERGIC) (Ge et al., 2013; Tan et al., 2013), plasma membrane (Ravikumar et al., 2010), endoplasmic reticulum (ER) (Sanchez-Wandelmer et al., 2015), endosome (Longatti et al., 2012; Knævelsrud et al., 2013), and is sourced from the cargo itself such as the mitochondria (Yamashita and Kanki, 2017). However, it is most likely a combination of these that varies depending on the cellular context, and cargo carried (Kojima et al., 2021).

This initiation is a stepwise process with the initial activation of the unc-51-like autophagy activating kinase (ULK1/2) complex, which includes focal adhesion kinase family interacting protein of 200 kDa (FIP200 RB1CC1), autophagy-related protein 13 (Atg13), and autophagy-related protein 101(Atg101). The activity of this complex is inhibited by the mammalian target of rapamycin (mTORC1) or specifically mTOR complex 1 through phosphorylation of ULK1/2. mTORC1 is involved in cell growth and proliferation, and its downstream targets are eukaryotic initiation factor (eIF), 4E-binding protein 1 (4EBP1) and ribosomal S6 protein kinase 1 (SK61). Its activity is sensitive to stress signals, particularly nutrient deprivation, or chemical inhibition with rapamycin directly or indirectly *via* other molecules such as AMP-activated protein kinase alpha subunit (AMPKα) activation, which block mTORC1. All result in releasing the inhibitory effects of mTORC1 and facilitating autophagy activation through the ULK1/2 complex (Chan et al., 2009; Hosokawa et al., 2009; Alers et al., 2012; Shi et al., 2020). The activated ULK1/2 complex interacts with the phagophore and PI3K complex. This interaction between these complexes, and the phagophore is suggested to be maintained by autophagy-related protein 9 (Atg9). Atg9 is also responsible for all membrane trafficking to the growing autophagosome; furthermore, it has been observed that the activity of Atg9 is dependent on ULK1 and PI3K complexes (Figure 1) (Ravikumar et al., 2009; Orsi et al., 2012; Karanasios et al., 2016; Guardia et al., 2020; Mailler et al., 2021). The PI3K complex consists of beclin1, phosphatidylinositol 3-kinase catalytic subunit type 3 (VPS34 or PI3KC3), vacuolar protein sorting 15 (VPS15) and autophagy-related protein 14 (Atg14). Because of its activation, it recruits phosphatidylinositol 3-phosphate (PI3P) to enable the elongation of the autophagosome and allow binding to WD-repeat protein interacting with phosphoinositides (WIPI). This binding of WIPI facilitates the recruitment of the autophagy-related 16-like 1 (Atg16L) complex.

#### Phagophore elongation and maturation of the autophagosome

Following phagophore formation is phagophore elongation, which involves many molecules and interactions, including the Atg12 conjugating system and two ubiquitin systems (Bhutia et al., 2013). The ubiquitin-like E1 enzyme (Atg7) and the ubiquitin-like E2 enzyme (Atg10) covalently conjugate Atg5 to Atg12 (Ravikumar et al., 2009; Li et al., 2017). The conjugated Atg5-Atg12 also non-covalently binds with Atg16L to form the Atg5-Atg12-Atg16L complex (Figure 1) (Li et al., 2017).

Autophagosome formation begins when LC3 is cleaved by Atg4 to produce LC3-I (Kabeya et al., 2000; Ravikumar et al., 2009; Bhutia et al., 2013). Next, the ubiquitin-like E1 enzyme Atg7 conjugates LC3-I to the lipid phosphatidylethanolamine (PE) to form LC3-II in a second ubiquitin system. LC3-II is the only Atg member to be in direct contact with the membrane of the autophagosome, and thus levels indicate the number of autophagosomes, where increased levels indicate more autophagosomes and *vice versa* (Kabeya et al., 2000; Ravikumar et al., 2009). Finally, the autophagosome completes its maturation and fuses closed. The removal of PI3P and Atg’s from the outer surface coincides, but the exact mechanisms or relationship of this final step in maturation is yet to be elucidated in the mammalian system, but in yeast, it is facilitated by Atg4 (Galluzzi et al., 2017). Evidence suggests that Atg12 and LC3-I conjugation are the leading mechanisms behind autophagosome maturation (Figure 1) (Ravikumar et al., 2009; Li et al., 2017).

#### Autophagosome-lysosome fusion

The mature autophagosome fuses with the lysosome to create the autolysosome (Figure 1) (Lőrincz and Juhász, 2020). This process begins when motor and coupling proteins like dynein, kinesins (KIFs 1-2), Rab7, and Arl8 facilitate the movement and contact between the two organelles (Harada et al., 1998; Brown et al., 2005; Korolchuk et al., 2011; Rosa-Ferreira and Munro, 2011; Xing et al., 2021a). Once in contact, they are tethered to each other with protein complexes that bind to GTPases present on the surface of each organelle. Homotypic fusion and vacuole protein sorting (HOPS) are proteins essential to this tethering process (Bröcker et al., 2012). It can tether the two organelles together as it contains two binding subunits (VPS41 and VPS39) on each end that recognise and bind to various proteins in both the autophagosome and lysosome (Bröcker et al., 2012). Proteins binding to the HOPS complex include syntaxin 17 (STX17) on the autophagosome, the GTPase Arl8b on the lysosome, and the GTPase Rab7 adaptor proteins PLEKHM1 and RILP which are present on both organelles (Jiang et al., 2014; Khatter et al., 2015; McEwan et al., 2015; van der Kant et al., 2015; Lőrincz and Juhász, 2020). Once tethered, the SNARE family of proteins mediates the fusion of the organelles. The major players are the lysosomal R-SNARE proteins VAMP8/VAMP7 and the autophagosome Q-SNARE proteins STX17 and SNAP-29 (Jiang et al., 2014; Saleeb et al., 2019). The regulation of this activity is shown to increase when UV radiation resistance-associated (UVRAG) binds the PI3K complex, or conversely, the binding of rubicon (RBCN) inhibits it (Galluzzi et al., 2017; Xing et al., 2021b; Tremel et al., 2021). The final step in the degradation process is the release of hydrolase enzymes from the lysosome into the autophagosome, where the contents are broken down to their constituents and then exported through the autolysosome membrane to the cytosol. Then the autolysosome is degraded, and the lysosomal components are stored for the development of lysosomes at a later stage (Noda et al., 2009; Chen and Yu, 2017; Nakamura and Yoshimori, 2017; Reggiori and Ungermann, 2017; Yu et al., 2018; Shrestha et al., 2020; Gatica et al., 2021).

### Autophagy regulation

The regulation of autophagy is dependent on nutrient signalling pathways, including the mammalian target of rapamycin (mTOR) and AMP-activated protein kinase (AMPK) (He and Klionsky, 2009; Jung et al., 2010). Whilst other pathways and molecules have been implicated in the regulation of autophagy, these two are commonly studied and understood.

### Mammalian target of rapamycin (mTOR)

**FIGURE 2:**
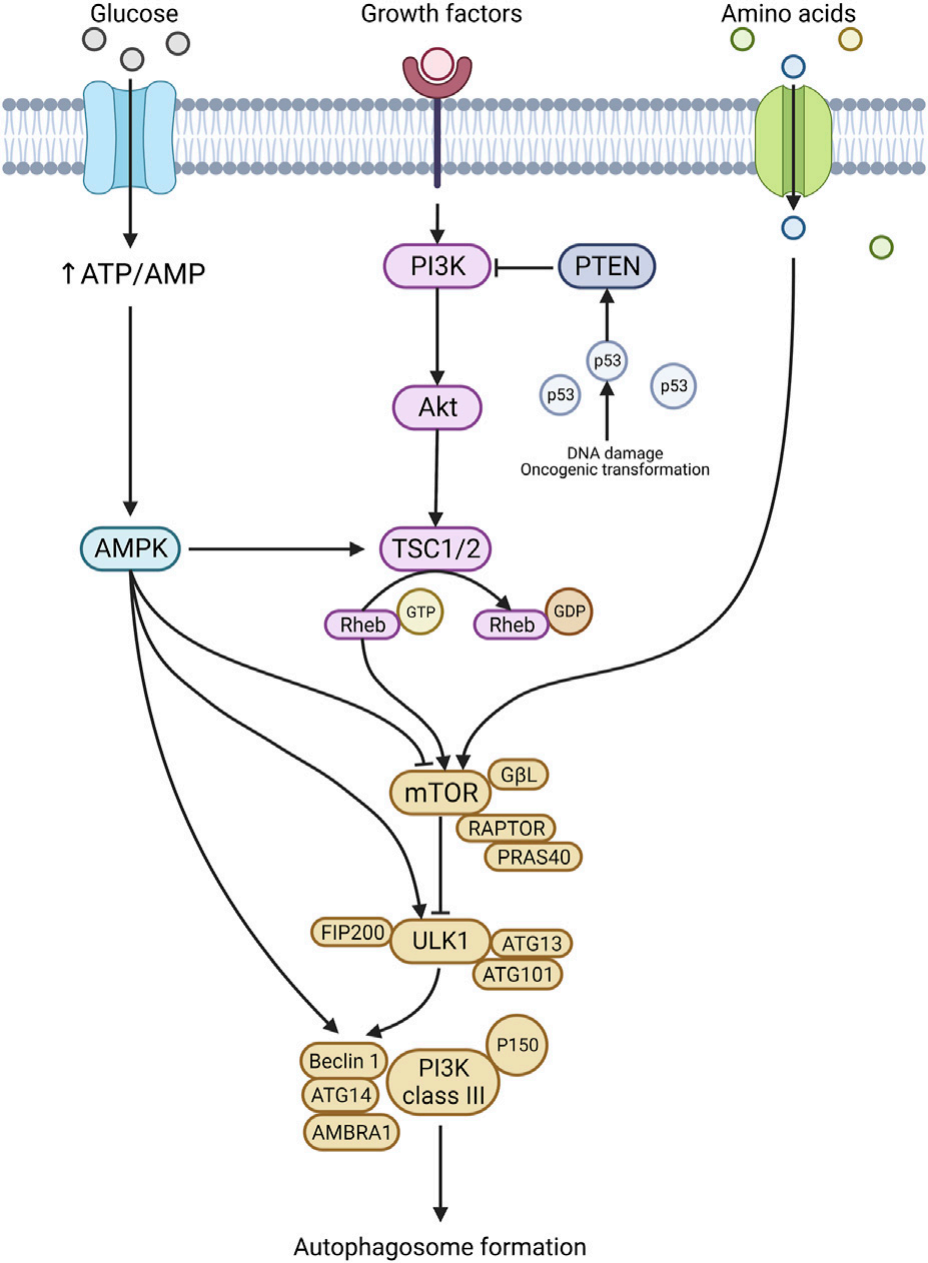
Autophagy regulation. Autophagy initiation is stimulated in response to nutritional stress, genotoxic stress, and various other stresses. A prominent player in autophagy regulation is the mTOR pathway and mTORC1. Once activated, mTORC1 stimulates the ULK1 complex and the downstream signalling cascade results in autophagy formation. The mTORC1 complex is stimulated by several upstream targets, including the PTEN/PI3K/Akt axis, amino acids, and AMPK. In addition**,** AMPK can also directly stimulate ULK1 and Beclin 1, leading to autophagosome formation. Created with BioRender.com.

The mTOR pathway is activated through nutrient starvation, cellular/environmental stresses (hypoxia, osmotic stress, heat shock, ROS, DNA damage, ER stress, decreased trophic factors) and reduced growth factor signalling (Jung et al., 2010). These stimuli start an autophagy signalling cascade by inhibiting mTORC1 (protein complex consisting of mTOR, GβL, RAPTOR, and PRAS40) (Jung et al., 2010). mTORC1 normally inhibits the ULK1 complex; thus, by suppressing mTORC1, the ULK1 complex becomes stimulated (Figure 2) (Ganley et al., 2009; Jung et al., 2009; Kim et al., 2011). The ULK1 complex activates downstream signalling complexes upon stimulation, leading to phagophore formation (Chan et al., 2009). Additionally, the PTEN/PI3K/Akt pathway is a known negative regulator of mTOR. Upon activation *via* factors like insulin/IGF, PI3K phosphorylates and stimulates Akt. Akt activation then facilitates the phosphorylation of TSC2 and PRAS40, activating mTOR (Figure 2) (Memmott and Dennis, 2009). PTEN, when activated, acts to inhibit PI3K and is controlled upstream by p53. Thus, oncogenic transformations and genotoxic stress, which stimulate p53, can also inhibit mTOR (Figure 2) (Levine and Abrams, 2008). In many cancers, mTORC1 is observed to be upregulated, and this is often a consequence of mutations or hyperactivation of upstream regulators Akt, PI3K and RAS or suppression of inhibitory regulators such as liver kinase B1 (LKB1), the phosphatase tensin homolog deleted on chromosome 10 (PTEN) and tuberous sclerosis proteins 1 and 2 (TSC1/2).

#### AMP-activated protein kinase (AMPK)

The AMPK pathway is the crucial homeostatic regulator of ATP levels in the cell and responds to mitochondrial stress (Herzig and Shaw, 2018). The AMPK signalling pathway can initiate autophagy by influencing numerous proteins, including mTORC1, ULK1, and Beclin1 (Figure 2) (Wang et al., 2022). Under glucose deprivation, AMPK facilitates ULK1 and Beclin1 activation *via* phosphorylation at specific sites (Chan et al., 2009; Kim et al., 2011; Zhang et al., 2016). ULK1 and Beclin1 are essential molecules for canonical autophagy initiation, and thus AMPKs’ ability to stimulate these molecules makes it a key regulator. AMPK also indirectly activates ULK1 through mTOR. In response to starvation, AMPK phosphorylates raptor, a key regulator of mTORC1 (Gwinn et al., 2008). Upon phosphorylation, raptor, a family of proteins known as 14-3-3, binds to and inactivates mTORC1; this subsequently activates the downstream autophagy initiation pathways (Figure 2) (Gwinn et al., 2008; Lee et al., 2010).

## Autophagy and therapeutic resistance in cancer

Cancer resistance to therapeutics has been an ongoing issue since the inception of cancer treatment. Enduring efforts have discovered numerous biological alterations in resistant cancers, a more recent one being that of autophagy (Paglin et al., 2001; Sotelo et al., 2006; Abedin et al., 2007; Amaravadi et al., 2007; Carew et al., 2007; Sun et al., 2011; Zou et al., 2012). In recent years, the development and discovery of autophagic inhibitors have shown promising results in decreasing therapeutic resistance in numerous cancers (Li et al., 2017; Mele et al., 2020). Underpinning this is mounting evidence demonstrating that the genetic ablation (including knockdown and knockout) of numerous autophagic genes increases therapeutic sensitivity (Chen et al., 2014a; Encouse et al., 2014; Kriel et al., 2018). However, its potential in helping cancer treatment extends past this, with evidence suggesting that it helps decrease proliferation, migration, and invasion (Medici et al., 2008; Gao et al., 2010; Catalano et al., 2015; Mowers et al., 2017; Colella et al., 2019).

Furthermore, studies using knockdowns of Atg’s and autophagy inhibitors have demonstrated that autophagy inhibition increases the efficacy of a range of therapeutics (Yang et al., 2011). The re-sensitising effect that autophagy inhibitors have had on cancer cell lines and mouse models has been shown in a plethora of cancers, including, BRAF-mutant brain cancers and thyroid cancers, bladder cancer, non-small-cell lung cancer (NSCLC) and ALK-positive NSCLC (Ji et al., 2014; Levy et al., 2014; Ma et al., 2014; Chen and Shi, 2016; Wang et al., 2016; Kang et al., 2017). Table 1 summarises some of the available research on current autophagy inhibitors’ effect on therapeutic resistance in cancer.

**TABLE 1:** Summary of the effect autophagy modulators have on therapeutic resistance in cancer.

Intervention	Mode of action	Effect on autophagy	Cancer	Treatments	Effect on resistance	References
CQ	Autophagosome-lysosome fusion inhibitor and/or lysosomal lumen alkalizer	Inhibits	CML	SAHA	Decreases	Carew et al. (2007)
			BRAF-mutant brain cancer	Vemurafenib, Trametinib	Decreases	Levy et al. (2014)
			Colon	Oxaliplatin, BEV	Decreases	Selvakumaran et al. (2013)
			NSCLC	Crizotinib, Erlotinib, Lidamycin, Ceritinib, Icotinib	Decreases, no change	Zou et al. (2013), Ji et al. (2014), Liu et al. (2014), Datta et al. (2019), Schläfli et al. (2021), Lyu et al. (2022)
			GBM	BEV, TMZ, Curcumin	Decreases	Hu et al. (2012), Encouse et al. (2014), Zanotto-Filho et al. (2015), Huang et al. (2018b)
			B cell lymphoma	Tamoxifen, Cyclophosphamide	Decreases	Amaravadi et al. (2007)
			Cutaneous squamous cell carcinoma	Luteolin	Decreases	Verschooten et al. (2012)
			Ovarian	Cisplatin	Decreases	Yan et al. (2019), Hwang et al. (2020)
			Prostate	Enzalutamide, AZD5363, Apalutamide	Decreases	Lamoureux and Zoubeidi (2013), Nguyen et al. (2014), Eberli et al. (2020)
			HCC	Sal	Decreases	Jiang et al. (2023)
			Bladder	Lapatinib, Gefitinib	Decreases	Kang et al. (2017)
3-MA	Autophagosome formation inhibitor; PI3K class III and class I inhibitor; binds to ATP binding site of PI3K	Inhibits	Bladder	Lapatinib, Gefitinib	Decreases	Kang et al. (2017)
			CML	SAHA	Decreases	Carew et al. (2007)
			GBM	TMZ, Curcumin	Decreases, increases	Kanzawa et al. (2004), Encouse et al. (2014), Filippi-Chiela et al. (2015), Zanotto-Filho et al. (2015)
			Head and Neck	RITA	Decreases	Shin et al. (2017)
			Prostate	AZD5363, Apalutamide	Decreases	Lamoureux and Zoubeidi (2013), Eberli et al. (2020)
			NSCLC	Icotinib	No change	Lyu et al. (2022)
			Gastric	Epirubicin, Cisplatin, 5-FU	Decreases	Ge et al. (2014)
			Breast	Camptothecin	Decreases	Abedin et al. (2007)
			Tongue	Erlotinib	No change	huang and liu (2016)
BafA1	Autophagosome-lysosome fusion inhibitor; blocks vacuolar-type H (+)-V-ATPase machinery	Inhibits	Bladder	Lapatinib, Gefitinib	Decreases	Kang et al. (2017)
			GBM	TMZ	Decreases	Kanzawa et al. (2004), Filippi-Chiela et al. (2015)
			NSCLC	Ceritinib, Icotinib	Decreases	Schläfli et al. (2021), Lyu et al. (2022)
			Breast	Camptothecin	Decreases	Abedin et al. (2007)
			prostate	AZD5363	Decreases	Lamoureux and Zoubeidi (2013)
HCQ	Autophagosome-lysosome fusion inhibitor and/or lysosomal lumen alkalizer	Inhibits	BRAF-mutant thyroid	Vemurafenib	Decreases	Wang et al. (2016)
			Melanoma	PLX4720	Decreases	Ma et al. (2014)
			GBM	TMZ, BEV	Decreases, no change	Buccarelli et al. (2018), Kriel et al. (2018), Liu et al. (2019)
			tongue	Erlotinib	Decreases	huang and liu (2016)
			Lung	Trametinib	Decreases	Bhatt et al. (2023)
Rapamycin	Autophagosome formation inhibitor; mTOR inhibitor; forms a complex with FKBP12 which binds to and inhibits mTORC1	Activates	GBM	TMZ, Radiation	Increases, decreases	Zhuang et al. (2011), Filippi-Chiela et al. (2015), Kriel et al. (2018)
			Osteosarcoma	Gemcitabine	Decreases	Ando et al. (2020)
			Tongue	Erlotinib	Increases	huang and liu (2016)
			Breast	Resveratrol, Doxycycline	Decreases, no change	Alayev et al. (2015), Dankó et al. (2021)
ACY-241	Unknown activator of autophagy	Activates	Pancreatic	Erlotinib	Increases	Park et al. (2021)
Clomipramine	Autophagosome-lysosome fusion inhibitor of unknown mechanism	Inhibits	Prostate	Enzalutamide	Decreases	Nguyen et al. (2014)
Metformin	Autophagosome formation inhibitor; AMPK activation/mTOR inhibition *via* increased phosphorylation	Inhibits	Prostate	Enzalutamide	Decreases	Nguyen et al. (2014)
IL-6	Activator of autophagosome formation; regulates PI3KC3 complex formation *via* phosphorylation of BECN11	Activates	Colorectal	Oxaliplatin, 5-FU	Increases	Hu et al. (2021)
Lys05	Autophagosome-lysosome fusion inhibitor and/or lysosomal lumen alkalize	Inhibits	Melanoma	PLX4720	Decreases	Ma et al. (2014)
VPS34-IN1	Autophagosome formation inhibitor; PI3K class III inhibitor; potentially binds to the PtdIns(3)P-binding PX domain	Inhibits	NSCLC	Ceritinib	Decreases	Schläfli et al. (2021)
Wortmannin	Autophagosome formation inhibitor; targets and inhibits all PI3Ks	Inhibits	Breast	Camptothecin	Decreases	Abedin et al. (2007)

GBM, glioblastoma; NSCLC, non-small cell lung cancer; CML, chronic myeloid leukaemia; TMZ, temozolomide; 3-MA; 3-Methyladenine, HCQ; hydroxychloroquine, CQ; chloroquine, BafA1; Bafilomycin A1, SAHA; suberoylanilide hydroxamic acid, IL-6; interleukin 6, HCC, hepatocellular carcinoma, Sal; Salidroside, 5-FU; 5-Fluorouracil.

Research demonstrating the effect of autophagy inhibition on therapeutic efficacy is readily available; however, the mechanism underlying autophagy’s involvement is still poorly understood. The current knowledge gap contributes to the lack of robust autophagic inhibitors in development.

## Pharmacological activators of autophagy

To be comprehensive and give insight into the canonical pathway, autophagic activation has to be considered. Classically autophagy is induced by nutritional deprivation and can be replicated in pre-clinical studies with glucose withdrawal and amino acid deprivation (Klionsky et al., 2021). Several pharmacological agents can induce autophagy by inhibiting negative or activating positive regulators within the canonical pathways. These included AMPK activation through agents such as metformin, quercetin, resveratrol, and mTOR inhibition through rapamycin (Figure 1).

### Rapamycin

Rapamycin is an antifungal metabolite produced by *Streptomyces* Hygroscopicus, which is a bacterium that resides in soil. It was observed that rapamycin binds to12-kDa FK506-binding protein (FKBP12) to form a complex that binds to mTORC1 and inhibits its activity. It has been shown to have activity against mTORC2 with prolonged use but does have greater specificity for mTORC1 (Li et al., 2014). mTORC1 directly controls protein synthesis and regulates metabolic pathways, particularly glycolysis. Rapamycin inhibition of mTORC1 to induce autophagy in yeast and most mammalian cells has resulted in it being used extensively in studying the mechanisms and actions of autophagy (Nyfeler et al., 2011; Klionsky et al., 2021).

Therefore, the potential for inhibiting mTORC1 with rapamycin was a promising cancer therapeutic; however, it was deemed not feasible due to solubility issues and its pharmacokinetics. Since then, there has been the development of analogues with improved solubility and pharmacology, which have included temsirolimus and everolimus. These analogues are approved by the Food and Drug Administration (FDA) in the USA for the treatment of angiomyolipomas, HER2-negative breast cancers, pancreatic neuroendocrine tumours, renal cell carcinomas and subependymal giant cell astrocytoma, and are being tested in numerous cancers in various clinical trial regime (Roskoski, 2019). However, despite improving the targeting of mTORC1 with these rapamycin analogues, their efficacy has overall been disappointing due to resistance and off-target effects (Hua et al., 2019).

### Pharmacological inhibitors of autophagy

Currently, two main classes of autophagy inhibitors will be the focus of this review. These are PI3K inhibitors that stop autophagosome formation and lysosomal inhibitors that prevent proper lysosome acidification (Figure 1).

#### PI3K inhibitors

PI3K inhibitors comprise chemical compounds that inhibit the PI3K complex and, thus, autophagy initiation in the canonical pathway, as previously discussed (Figure 1). The PI3K family is divided into classes I, II and III, with numerous isoforms within classes I and II. PI3K class I isoforms form part of the Akt mTOR axis and regulate growth, metabolism, cellular movement, and protein synthesis. PI3K class II isoforms are involved in endocytosis, mitosis, and cell migration. The class III enzyme is involved in autophagy and extra vesicle trafficking. Thus ideally, PI3K inhibitors would be targeted towards class III enzymes; however, until recently, inhibitors have lacked this specificity. The most common inhibitors include 3-Methyladenine (3-MA), Wortmannin and LY294002 (Arcaro and Wymann, 1993; Powis et al., 1994; Vlahos et al., 1994; Wu et al., 2010).

One of the most commonly used autophagic inhibitors in pre-clinical studies over the last decade has been that of 3-MA. 3-MA inhibits VPS34 by binding to its ATP binding site. 3-MA inhibits the class III PI3K only transiently whilst blocking class I PI3K more persistently (Wu et al., 2010). In addition, it only suppresses autophagy under starvation conditions and in fact promotes autophagy in complete media, similar to rapamycin and the suppression of mTOR (Wu et al., 2010). Therefore, further research using 3-MA as an autophagy inhibitor should potentially be discontinued due to this uncertainty and the availability of more selective autophagy inhibitors. In addition, its poor solubility has severely limited its use in several settings, including clinical studies. However, derivatives of 3-MA have been developed and have improved solubility and specificity to class III PI3K (Wu et al., 2013). Thus, these derivatives should be used going forward.

A few recent studies have proven efficacious in treating colorectal cancer cells in hypoxic conditions with 3-MA causing apoptosis (Dong et al., 2019). Another has shown that treatment in head and neck cancers with reactivation of p53 and induction of tumour cell apoptosis (RITA). The treatment potential of RITA has been limited to date due to resistance which can be overcome with the addition of 3-MA (Shin et al., 2017). Curiously, both studies presented LC3 levels to represent autophagy but did not show any PI3K class I activity to ascertain if this pathway had a role. Additionally, a study that examined the efficacy of various PI3K inhibitors; 3-MA, the newer derivative Autophagy inhibitor IV, more commonly referred to as Compound 18 (Cpd18) (Wu et al., 2013), SAR-405 and lysosomal inhibitors demonstrated that 3-MA was the most effective at reducing cell viability across cell lines compared to Cpd18, SAR-405 and lysosomal inhibitors. However, it was shown that 3-MA induced DNA damage resulting in subsequent cell death. It was also shown that 3-MA had a minimal inhibitory effect on PI3K class I downstream targets. Moreover, both Cpd18 and SAR405 were found to have no effect on the PI3K class I targets (Chicote et al., 2020).

Alternative drugs to 3-MA include Wortmannin and LY294002. Wortmannin transiently blocks PI3K class I but persistently blocks PI3K class III (Powis et al., 1994; Wu et al., 2010) and LY294002, an analogue of the naturally occurring compound Quercetin, specifically targets and inhibits PI3K activity (Vlahos et al., 1994).

SAR-405 is a first-in-class ATP-competitive inhibitor of VPS34 and was identified through high throughput small molecule screening. Its activity is specific to VPS34, inhibiting autophagy and late endosome to lysosome trafficking (Pasquier, 2015; Bago et al., 2016). Interestingly, it has been shown that the SAR-405 IC50 significantly decreases when an mTOR inhibitor induces autophagy. Therefore, a study into the combination of SAR-405 and mTOR inhibitor everolimus showed increased cytotoxicity in renal cancer cell lines (Ronan et al., 2014; Pasquier, 2015). More recently, it has been shown to induce inflammation within the tumour and provide an environment for the successful use of immunotherapeutic agents *in vivo* in melanoma and colorectal cancer models (Noman et al., 2020). In addition, evidence shows that SAR-405 ameliorates radiotherapy-induced mitophagy and improves tumour refraction when radiotherapy is combined with SAR-405 in head and neck cancer *in vivo* (Lee et al., 2021).

VPS34-IN1 is a specific and potent VPS34 inhibitor, and its mechanisms of action are suggested to be through its PtdIns(3)P-binding PX domain but are yet to be fully elucidated (Bago et al., 2014). A recent study showed improved efficacy of Ceritinib with VPS34-IN1 in treating non-small cell lung cancer cells and reduced cell survival (Schläfli et al., 2021). Ceritinib is an anaplastic lymphoma kinase (ALK) inhibitor which activates several signalling cascades resulting in the inhibition of PI3K. It is this inhibition of PI3K that reduces proliferation, tumour growth and induces apoptotic cell death. The additive effect that VPS34-IN1 has on these cells suggests that Ceritinib may only be targeting PI3K class I and class II.

It was also suggested that VPS34-IN1 and SAR-405 offered more significant treatment potential due to their specificity (Schläfli et al., 2021). Recently, VPS34-IN1 has been shown to have promising effects in acute myeloid leukaemia (AML) cells, with increased cell death observed in response to treatment (Meunier et al., 2020).

##### Lysosomal inhibitors

Lysosomal inhibitors include numerous compounds that alter the pH of the lysosome thereby making the function of the enzymes that rely on this defunct. Common lysosomal inhibitors include Bafilomycin A1 (BafA1), chloroquine (CQ), and hydroxychloroquine (HCQ).

BafA1, a vacuolar-type H (+)-V-ATPase inhibitor, is an antibiotic that inhibits subunit c of the V-ATPase machinery (Wang et al., 2021). V-ATPases are a type of proton pump essential for maintaining the acidic environment in the lysosome, which is required for proper functioning and activation of degradative enzymes (Mauvezin and Neufeld, 2015). BafA1 prevents both acidifications of the lysosome and autophagosome-autolysosome fusion by disrupting the function of the V-ATPases (Yamamoto et al., 1998). It has also been shown to be a potassium carrier to mitochondria, resulting in mitochondrial swelling and dysfunction; this activity was observed at nanomolar concentrations (Teplova et al., 2007). It has been suggested that the use of BafA1 is not feasible due to this toxicity, although interestingly, claims of toxicities have not been supported by data, and on the contrary, there are studies, at least in leukaemia, that have shown no toxicity *in vivo* (Yan et al., 2016; Li et al., 2020). It is proposed that this suggested toxicity is possibly related to off-target effects at higher concentrations. However, as the current evidence demonstrates this off-target toxicity is not observed at lower concentrations where BafA1 has been shown to be effective in *in vivo* models for a variety of cancer types, this still maybe worth exploring (Pivtoraiko et al., 2010; Yuan et al., 2015; Yan et al., 2016; Fitzwalter et al., 2018). Despite this, to date its use in humans has not been approved.

CQ and its analogue HCQ are lysosomal lumen alkalises. They are lysosomotropic and accumulate within acidic vessels, particularly the lysosome, where their weak base formula reduces lysosome acidity and function, although the exact mechanism is yet to be elucidated. Other research suggests that they affect the lysosome and autophagosome-lysosome fusion (Shingu et al., 2009; Mauthe et al., 2018). Although hydroxychloroquine is approved by the Therapeutic Goods Administration (TGA) for the treatment of rheumatoid arthritis, systemic lupus erythematosus, discoid lupus erythematous and malaria, its use as a co-treatment in cancer may not be viable due to the high concentrations required to inhibit autophagy and the detrimental symptoms that arise because of this (arrhythmias, myopathy, cardiovascular cytotoxicity) (Rosenfeld et al., 2014; Al-Bari, 2015). One potential remedy to that is using a more potent analogue, several of which have already been developed, the most notable being Lys01 and Lys05 (McAfee et al., 2012).

A systematic review and meta-analysis of all cancer trials that have been undertaken using chloroquine or hydroxychloroquine for cancer treatment were analysed to determine efficacy. The review assessed the use combined with gemcitabine, doxorubicin, radiation, temozolomide and single therapy with hydroxychloroquine. Cancers included were glioblastoma, non-Hodgkin’s lymphoma, pancreatic, metastatic non-small cell lung cancer, and metastatic breast cancer. The meta-analysis demonstrated that the overall response rate was significantly higher with the inclusion of hydroxychloroquine or chloroquine than without (Xu et al., 2018). However, stress exerted on the ER and Golgi by HCQ and CQ may also have contributed to some of the clinical results rather than just autophagic inhibition alone (Mauthe et al., 2018).

#### Promising inhibitors of autophagy initiation molecules

Current autophagy inhibitors lack specificity, and historically, very few inhibitors have been shown to directly impact the Atg’s and other autophagy markers, including the ULKs, LC3s, RB1CC1, and GABARAP (Akin et al., 2014). Recently, NSC185058 has been demonstrated to be an agonist of ATG4B and LC3B lipidation (Akin et al., 2014). Computational research has suggested that it exerts this effect by binding to the pocket of ATG4B required for its proteolytic activity (Akin et al., 2014). Additionally, Akin and colleagues have demonstrated in osteosarcoma that cell viability and tumour size were significantly reduced upon the addition of NSC185058. They have established it to be a potent stand-alone treatment (Akin et al., 2014). Aside from NSC185058, several other drugs, including S130, 4-28, LV-320, and S068, have been screened, but very little has been done to confirm their *in vivo* efficacy (Cleenewerck et al., 2016; Bosc et al., 2018; Agrotis and Ketteler, 2019; Fu et al., 2019). It has also been suggested that targeting other Atg’s such as Atg7 and Atg5, may not be appropriate as knockout studies have demonstrated an increase in cytotoxicity, mortality in mice, reduced lifespan, and neurodegeneration, to name a few deleterious impacts (Karsli-Uzunbas et al., 2014; Kuma et al., 2017; Yoshii et al., 2017).

Lazarus et al. (Lazarus et al., 2015) pioneered the initial identification of the ULK1 structure, which aided in identifying and designing targeted inhibitors toward ULK1. The most recent was Martin et al., who performed a small molecule screen and identified several potential inhibitors of ULK1, which was later confirmed by further *in vitro* analysis (Martin et al., 2018). The results also indicate their efficacy against ULK2 which, as expected, would improve its inhibitory effects on autophagy activity. Their activity was examined further to reduce it to one specific inhibitor, ULK-101, with efficacy at the low nanomolar ranges in non-small cell lung cancer (Martin et al., 2018). Furthermore, in line with our unpublished findings and others, KRAS mutant cancer is more susceptible to autophagic inhibition in the context of lung cancer, particularly in a nutrient-deprived microenvironment. In addition, ULK1 inhibition resulted in the cessation of autophagosome formation/maturation demonstrating its specificity of action and promise as a future therapeutic (Maria et al., 2020).

### Clinical trials targeting autophagy

The chemical modulation of autophagy in cancer as an adjuvant therapy prospect is well-established. There have been over 100 clinical trials and more than 30 are currently being undertaken to explicitly determine the efficacy and appropriateness of mainly autophagic inhibitors, (with a few in autophagy activators) in cancer treatment. The aim in most of these trials is to increase the effectiveness of frontline treatment by limiting resistance, as summarised in Table 2. However, nearly all of the trials are utilising hydroxychloroquine or chloroquine. As discussed earlier, these compounds have other mechanisms of action in conjunction with autophagy inhibition. Additionally, autophagy inhibition is often only achieved with high concentrations of HCQ, and this consequently leads to a multitude of adverse side-effects. In previous trials with HCQ treatment where patients’ samples were collected, there was no evidence to demonstrate the expected inhibition of autophagy (NCT03344172). Therefore, the translation from bench to bedside is being impeded by the lack of available specific and potent autophagic inhibitors. Response to the clinical demand has led to the trialling of non-specific alternatives that do not truly reflect the therapeutic benefit of autophagic inhibition in cancer treatment.

**TABLE 2:** Summary of the current and active clinical trials implementing autophagy modulation.

Clinical trial phase	Intervention	Treatment dose	Action on autophagy	Condition	Status	NIH ClinicalTrials.gov REF No.
I/II	Hydroxychloroquine, Palbociclib, and Letrozole	To be defined	Inhibition	ER positive HER2 negative breast cancer	Active	NCT03774472
I/II	mFOLFIRINOX With Perioperative Oral Hydroxychloroquine	Hydroxychloroquine- escalated from 400mg to 1200 mg	Inhibition	Pancreatic Adenocarcinoma (resectable)	Recruiting	NCT04911816
II	Sorafenib and Hydroxychloroquine	Sorafenib-400 mg daily	Inhibition	Hepatocellular Cancer	Recruiting	NCT03037437
		Hydroxychloroquine −400 mg daily				
II	Cobimetinib (MEK Inhibitor), Atezolizumab (Immune Checkpoint Blockade), Hydroxychloroquine	As per cycle Cobimetinib 40–60 mg daily	Inhibition	Gastrointestinal, pancreatic, and agnostic cancer (specifically KRAS-mutated advanced malignancies)	Active	NCT04214418
		Hydroxychloroquine −600 mg twice daily				
		Atezolizumab-840 mg day 1 and 15				
II	Hydroxychloroquine Encorafenib and Cetuximab or Panitumumab	As per cycle	Inhibition	Stage IV Colorectal (BRAF V600E)	Recruiting	NCT05576896
		Hydroxychloroquine-Not stated				
		Encorafenib −300 mg daily				
		Cetuximab-400 mg/m^2^, 250 mg/m^2^				
		Panitumumab-Not stated				
II	LY3214996 with or without Hydroxychloroquine	To be defined	Inhibition	Pancreatic cancer (Metastatic)	Recruiting	NCT04386057
I/II	Hydroxychloroquine, nelfinavir, metformin, dasatinib and sirolimus	To be defined	Inhibition And Activation	Advanced solid tumours or Relapse prostate cancer	Recruiting	NCT05036226
I	Enzalutamide and Metformin Hydrochloride	To be defined	Activation	Hormone-resistant prostate cancer	Active	NCT02339168
I	MK2206 (AKT inhibitor) and Hydroxychloroquine	To be defined	Inhibition	Advanced solid tumours Melanoma, Prostate or Kidney	Active	NCT01480154
II	Trametinib (MEK Inhibitor) and Hydroxychloroquine	Trametinib -2 mg daily	Inhibition	Bile Tract Carcinoma (KRAS mutation refractory)	Recruiting	NCT04566133
		Hydroxychloroquine-600 mg twice daily				
I	Binimetinib and Hydroxychloroquine	To be defined	Inhibition	Metastatic Pancreatic cancer (KRAS mutation)	Recruiting	NCT04132505
I	TACE (trans arterial chemoembolization) Plus Axitinib and Hydroxychlorquine	TACE-4–8-week intervals	Inhibition	Metastatic colorectal cancer (liver dominant)	Recruiting	NCT04873895
		Axitinib- 5 mg twice daily				
		Hydroxychlorquine- 600 mg twice daily				
II	Autophagy Activation for the Alleviation of Cardiomyopathy Symptoms After Anthracycline Treatment (Carvedilol	To be defined	Activation	Breast Carcinoma	Active	NCT04190433
	Lisinopril			Hematopoietic and Lymphoid Cell Neoplasm		
	Pravastatin and Spironolactone)			Lymphoma		
				Sarcoma		
Early I	Quercetin, EGCG, metformin, zinc	Quercetin-500 mg daily	Activation	Metastatic Breast Cancer and Triple-negative Breast Cancer	Not yet recruiting	NCT05680662
		EGCG-300mg daily, Metformin −850 mg daily				
		Zinc-50 mg daily				
II	Paricalcitol and Hydroxychloroquine in Combination With Gemcitabine and Nab-Paclitaxel	To be defined	Inhibition	Advanced Pancreatic Cancer	Recruiting	NCT04524702
II	Ulixertinib in Combination With Hydroxychloroquine	Ulixertinib-50 mg twice daily	Inhibition	Advanced gastrointestinal malignancie (RAS mutation)	Recruiting	NCT05221320
		Hydroxychloroquine-600 mg twice daily				
I	CPI-613 (Devimistat) in Combination With Modified FOLFIRINOX Plus Bevacizumab	As per cycle	Activation	Metastatic Colorectal Cancer	Not recruiting yet	NCT05070104
		Devimistat CPI-613				
		250–1000mg/m2				
		Modified FFX				
		Irinotecan-50 mg/m^2^				
		Leucovorin-400mg/m2				
		Oxaliplatin-85mg/m2 5FU: 2400mg/m2 IV				
		Bevacizumab-5mg/kg				
II	Abemaciclib (CDK4/6 inhibitor) and Hydroxychloroquine	Abemaciclib −100 mg or 150 mg twice daily	Inhibition	Breast Cancer (residual disease)	Recruiting	NCT04523857
		Hydroxychloroquine-600 mg twice daily				
NA	Intermittent Fasting	5:2 Method (intermittent fasting regimen)	Activation	Chronic Lymphocytic Leukemia (CLL)	By invitation	NCT05708326
		16/8 Method (intermittent fast regimen)		Small Lymphocytic Lymphoma (SLL		
II	Dabrafenib and Trametinib With or Without Hydroxychloroquine	To be defined	Inhibition	Melanoma (Stage IIIC or IV BRAF V600 E/K)	Active	NCT04527549
II	Chemoimmunotherapy and fasting	Dietary Supplement: Control diet or Fasting-Like Approach anthracycline-taxane-carboplatin chemotherapy plus Pembrolizumab	Activation	Triple negative breast cancer	Not yet recruiting	NCT05763992
I	Sunitinib Malate and Hydroxychloroquine	To be defined	Inhibition	Advanced solid tumours (not responded to chemotherapy)	Active	NCT00813423
II	Avelumab or Hydroxychloroquine With or Without Palbociclib	As per cycle	Inhibition	Breast Cancer ER positive (disseminated tumour cells)	Recruiting	NCT04841148
		Avelumab-10 mg/kg				
		Hydroxychloroquine-600 mg twice daily				
		Palociclib-125 mg daily				
I	Carfilzomib in Combination With Cyclophosphamide and Etoposide	To be defined	Activation	Children with solid tumours (relapsed/refractory) or leukemia	Recruiting	NCT02512926
II	Ezurpimtrostat Autophagy Inhibitor in Association With Atezolizumab-Bevacizumab	As per cycle	Inhibition	Hepatocellular Carcinoma (unresectable)	Recruiting	NCT05448677
		Ezurpimtrostat-to be defined				
		Atezolizumab-1200 mg daily				
		Bevacizumab-15 mg/kg				
II	Chloroquine and Chemoradiation	Chloroquine-to be defined	Inhibition	Glioblastoma	Not yet recruiting	NCT02432417
		Temozolomide- 75 mg/m^2^				
		Radiation-30 fractions of 2 Gy (Gy)				
I/II	Gemcitabine, Docetaxel, and Hydroxychloroquine	To be defined	Inhibition	Osteosarcoma (recurrent or refractory)	Recruiting	NCT03598595
I/II	TN-TC11G (THC + CBD) Combination with Temozolomide and radiation	As per cycle	Activation	Glioblastoma	Not yet recruiting	NCT03529448
		TN-TC11G -to be defined				
		Temozolomide- 75 mg/m^2^, 150 mg/m^2^, 200 mg/m^2^				
		Radiation-1.8–2.0 Gy/day (total dose 58–60Gy)				
I/II	Dabrafenib, Trametinib and Hydroxychloroquine	To be defined	Inhibition	Glioma of the brain (low and high grade with BRAF aberration) Glioma of brain (low grade with neurofibromatosis type1)	Recruiting	NCT04201457

Information sourced from clinicaltrials.gov.

### Mechanism of autophagy underlying therapeutic resistance

**FIGURE 3:**
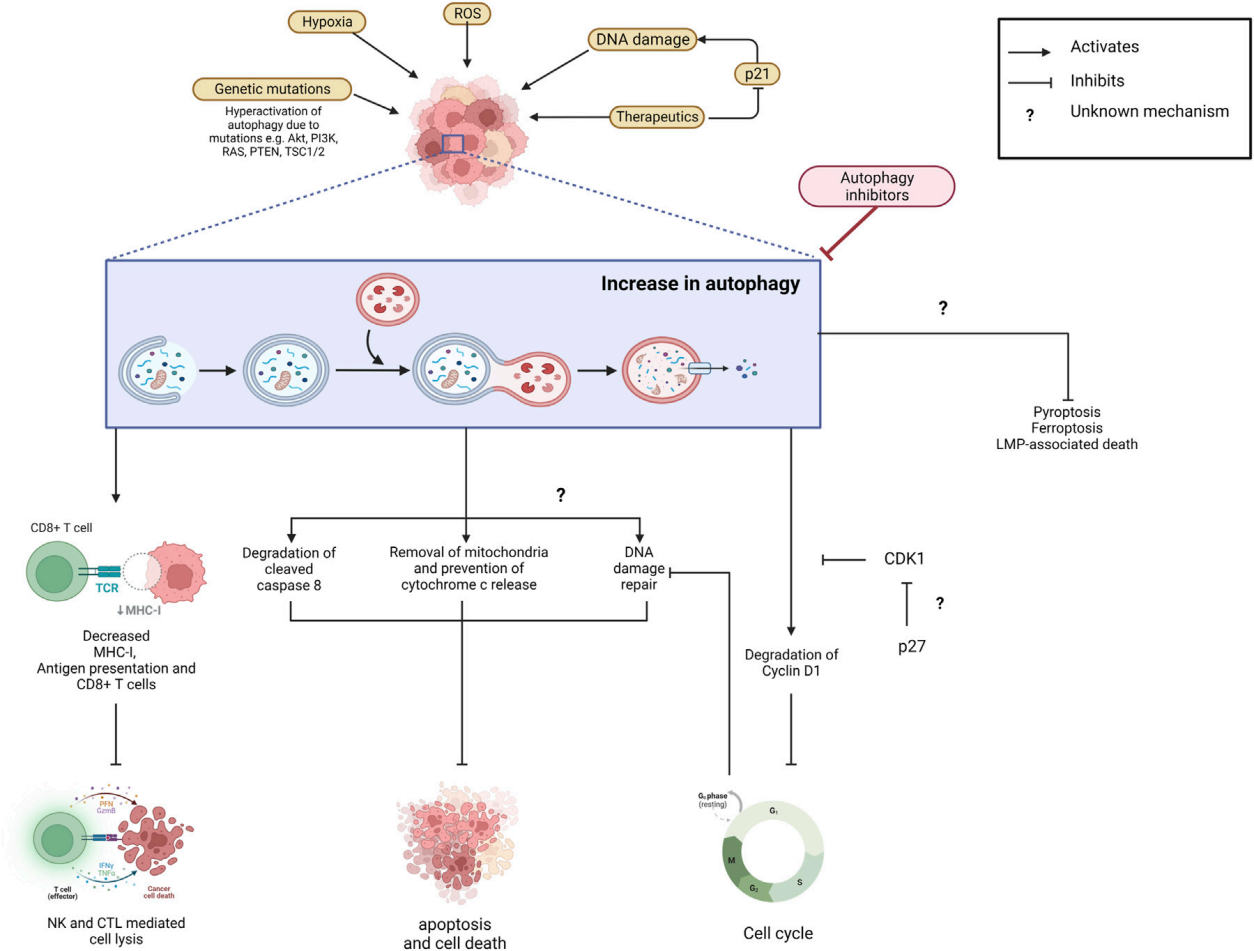
Increased autophagy prevents cancer cell elimination and contributes to therapeutic resistance. Autophagy is upregulated in cancer cells due to various microenvironmental stresses including exposure to therapeutics. Autophagy eliminates MHC-I and decreases antigen presentation and CD8^+^ T cells which inhibits NK- and CTL-mediated cell lysis and the thus the immune response. Apoptotic cell death is also reduced as autophagy facilitates mitochondria and cleaved caspase-8 degradation which prevents downstream signalling cascades. It also prevents death signalling cascades by actively promoting DNA damage repair. The mechanism behind this is unclear. Potentially, autophagy’s degradation of Cyclin D1 and thus cell cycle arrest allows for this. Its upregulation also prevents the Ferroptosis, Pyroptosis and LMP-associated death pathways through unclear mechanisms. Suppression of autophagy via inhibitors could prevent the degradation of components that would otherwise inhibit cell death pathways. Created with BioRender.com.

The exact mechanism by which autophagy affects therapeutic resistance is still unclear. Autophagy inhibition improves therapeutics’ efficacy with multiple actions ranging from DNA damage, cell cycle inhibition, and immune system recruitment (Figure 3). Autophagy inhibition can affect these vastly different therapeutics, suggesting that autophagy has multiple roles in resistance.

### Autophagy and DNA damage repair

Autophagy repairs DNA damage, so DNA-damaging agents that induce cell death *via* this pathway are less effective with increased autophagy. Thus, by inhibiting autophagy, damage to DNA can no longer be repaired, and the cells are destroyed. An example is the alkylating agent Temozolomide (TMZ), which is used in the treatment of Glioblastoma (GBM). This chemotherapeutic induces apoptotic cell death through the methylation of DNA residues resulting in DNA damage (Agarwala and Kirkwood, 2000). In addition, recent studies have suggested that autophagy is necessary for DNA Damage Response (DDR) (Liu et al., 2015; Qiang et al., 2016; Hewitt and Korolchuk, 2017). Thus, it has been proposed that due to this ability, autophagy increases the cell’s resistance to TMZ by increasing DDR (Liu et al., 2015). Numerous other therapeutics induce cell death *via* DNA damage, including Platinum (Cisplatin, Oxaliplatin), replication disrupting agents (Gemcitabine), and radio mimetics (Etoposide, Doxorubicin), which are common treatments for cancers including colon, breast, pancreatic, and lung as well as many more (Cheung-Ong et al., 2013; Woods and Turchi, 2013).

#### Autophagy and the cell cycle

Autophagy has been shown to regulate the cell cycle independent of its role in the DNA damage repair mechanisms as mentioned previously. Its role in cell cycle arrest is based on the understanding that it has a critical function in recycling regulatory components of the cell cycle. It is suggested that this degradation of cell cycle components is increased in cells being treated with chemotherapeutic agents targeted towards DNA and results in an upregulation of autophagy. Whereby autophagy stalls cell division allowing time for the cell to repair the DNA damage elicited by the treatment, but the exact mechanisms are yet to be elucidated (Galluzzi et al., 2015). In line with this, a preliminary clinical study demonstrated co-localisation between Beclin-1 and Cyclin D1 in glioblastoma patient’s primary and recurrent tumours treated with temozolomide and radiotherapy it was suggested that autophagy was removing Cyclin D1 (Luigi et al., 2016) which is an important regulator of cell cycle arrest. Furthermore, Cyclin D1 has also been observed to be targeted by autophagy in hepatocarcinoma (Wu et al., 2019). Interestingly, both studies suggest that autophagy’s role is cytoprotective although this may be reflective of the study limitations in glioblastoma as it has been shown in many other studies reviewed in this article that autophagy provides resistance against temozolomide in this context. Moreover, it is also suggested that the cytotoxic effects observed with rapamycin in the context of hepatocarcinoma may be resulting in a dysfunctional autophagy that then has elicited synthetic lethality in the cells rather than a functional protection (Wu et al., 2019).

The role of autophagy during the cell cycle has more recently been observed to vary at different stages, apart from induction, it is also suggested to be inhibited. The mitotic regulator Cyclin Dependent Kinase 1 (CDK1) was shown to inhibit autophagy directly during mitosis. This suppression is driven by binding of CDK1 to regulatory ATGs at sites usually bound by mTORC1 which facilitates the inhibition of autophagy (Odle et al., 2020). This is perplexing as CDK1 is commonly upregulated in cancer which would suggest increased autophagic inhibition would be expected. However, it is proposed that this inhibitory effect may be circumvented by p27 activity in some contexts therefore reinstating autophagic activity and is worth further investigation (Jung et al., 2018; Zhang et al., 2018).

Additionally, there is renewed interest in using CDKI inhibitors particularly in combination with immunotherapy to improve tumour cytotoxicity. Based on these previous findings there is a possibility autophagy would be inhibited, and within the context of treatment, may increase its efficacy. However, in this setting of immune therapy it may also have a potential to interfere with the role autophagy as has been shown in the T-cell response (Delamarre et al., 2005).

##### Autophagy and the immune system

Autophagy is implicated in regulating the innate and adaptive immune systems through its regulation of antigen presentation, cytokine release and T and B cell activity. It has been demonstrated to play a role in immune system suppression and tumour evasion of the immune system by various mechanisms. This includes degradation of MHC-I, suppression of antigen presentation and decreased CD4^+^ and CD8^+^ T cells (Baghdadi et al., 2013; Yamamoto et al., 2020). It has been well established that these factors are needed for recognition and elimination of cancer cells by cytotoxic T lymphocytes (CTL) or Natural Killer (NK) cells and the loss of these leads to the evasion of immune system (Yamamoto et al., 2020). Autophagy’s complex role in the immune system may be one explanation behind resistance towards immunotherapies. Immunotherapies are a class of therapies that promote the patient’s immune system and enhances the body’s ability to identify and destroy cancer cells. Autophagy’s role in decreasing NK- and CTL-mediated cell lysis may be a leading mechanism behind resistance to immunotherapies (Vessoni et al., 2013).

Thus, autophagy inhibition may make tumour cells more susceptible to the immune system but conversely may limit the capacity of the immune cells to function. This highlights the need for further studies to examine the effects of autophagic inhibition in this context to truly assess the potential of autophagy activity modulation and how it may improve current and design future immunotherapeutic strategies.

##### Autophagy and elimination of apoptotic death signals

Another potential mechanism by which autophagy can circumvent the efficacy of therapeutics is due to its inverse relationship with apoptosis. The upregulation of one appears to suppress the other. It has also been suggested that they may represent different sides of the same coin, but what exactly elicits one and not the other to control cell fate is unclear. Suppression of autophagy demonstrated a marked increase in therapeutic efficacy in resistant cells *via* an increase in apoptosis, as seen with imatinib in chronic myelogenous leukaemia (Carew et al., 2007). The mechanism behind this is unclear. There is potentially the involvement of p53, which facilitates several cascades that activate caspases and induces apoptosis. Furthermore, autophagy inhibition with CQ leads to an increase in p53 activation and apoptosis in lymphoma cells (Amaravadi et al., 2007).

Arguably the most influential group of proteins in classical apoptotic death signalling pathway are the BCL-2 apoptotic and antiapoptotic family members. The BCL-2 family members can be categorised into three classes. First there are the antiapoptotic family members (includes Bcl-2, Mcl-1, Bcl-XL), second the apoptotic members (includes BAK and BAX) and third the BH3 only proteins (includes BAD, BIK, BID) (Youle and Strasser, 2008). In brief, when the apoptotic family members are active, they stimulate cytochrome c release from the mitochondria which in turn activates caspases responsible for cell death (Leibowitz and Yu, 2010). The anti-apoptotic family members supress apoptotic members and are themselves supressed by the BH3 only proteins and other factors (e.g., UV, DNA damage, viruses) which leads to apoptosis (Youle and Strasser, 2008; Leibowitz and Yu, 2010).

Autophagy has been demonstrated to manipulate several members of this family, although its exact relationship to the anti-apoptotic and apoptotic members is unclear. One particularly important molecule in both apoptosis and autophagy is Beclin-1. As previously discussed Beclin-1 is a key member of the class III phosphatidylinositol −3 complex and is essential for autophagosome formation. It also forms a complex with BCL-2, and in this complex Beclin-1 was found to be inhibited from its function in autophagy (Pattingre et al., 2005). However, with stimuli that induce autophagy such as starvation, ROS, and hypoxia, Beclin1 dissociates from BCL2 (Pattingre et al., 2005; Wei et al., 2008; Bellot et al., 2009; Tang et al., 2010). Once dissociated, Beclin-1 can form the class III phosphatidylinositol −3 autophagosome formation complex, and BCL-2 continues to perform its antiapoptotic role.

In addition, Atg12 inactivates antiapoptotic members Bcl-2 and Mcl-1 by the binding of the BH3-like motif on Atg12 to the BH3-binding groove of BCL-2 family members (Rubinstein et al., 2011). Truncated Atg5 formed by the cleavage of ATG5 by calpains 1 and 2 is also able to induce apoptosis (Yousefi et al., 2006). Once Atg5 is truncated it translocates to the mitochondria where it facilitates the release of cytochrome c, and thus, stimulates apoptosis (Yousefi et al., 2006). It maybe postulated that the suppression of autophagy will result in these molecules being more readily available for apoptotic processes.

In contrast, autophagic inhibition increased cell death upon nutrient starvation in fibroblast cells. Although the mechanism is unclear it is believed that the apoptotic BAX and BAK molecules are involved as double knockout BAX^−/−^ BAK^−/−^ fibroblast cells had a reduced cell death upon nutrient starvation and autophagy inhibition (Boya et al., 2005). The contradiction may be explained by the context being a physiological setting compared to pathologic. As most research to date has demonstrated that autophagy is able to prevent apoptosis at the later stages. And is seen by its ability to degrade the mitochondria, thus preventing cytochrome c release, and degradation of the caspases.

Furthermore, cleaved caspase 8 is increased upon autophagy inhibition, and under normal conditions, the large subunit of caspase 8 is taken into the autophagosome and eliminated in the lysosome (Hou et al., 2010; Yan et al., 2019). Autophagy also potentially degrades cleaved caspase 3 as its presence increases in CQ-treated lymphoma cells (Amaravadi et al., 2007). In addition, several chemotherapies, including doxorubicin, trastuzumab and Sunitinib, can enlist apoptotic death signalling cascades because of the damaged mitochondria caused by the pharmacological compounds (Gharanei et al., 2013; Sarosiek et al., 2013; Westrate et al., 2014; Gilliam et al., 2016; Huang et al., 2018a; Gorini, 2018). Autophagy can circumvent the release of cytochrome c and the death cascade by pre-emptively degrading the mitochondria (Ravikumar et al., 2006; Colell et al., 2007). Thus, active autophagy can prevent apoptosis through this pathway, whilst suppression of autophagy can enable this pathway.

A recent study by Hwang *et al.* demonstrated that combining CQ and Cisplatin in ovarian cancer increased γH2Ax, a DNA damage marker, caspase 3, and phosphorylated ATM, and it does this through p21 suppression, which was suggested to induce autophagy (Hwang et al., 2020). These findings were further supported by Maheshwari et al. with their research confirming the negative regulation of autophagy by p21, which was observed to be facilitated by Akt (Maheshwari et al., 2022). Furthermore, these findings were observed in several cancer models, suggesting it is not context dependent (Maheshwari et al., 2022).

A recent study by Gremeke *et al. e*xplored the mechanisms of drug resistance in numerous cancers and found that the treatment induced autophagy activity. The results of this study highlight the complexity of targeting drug-refractory tumours such as NSCLC, whereby the mechanism of resistance in these cells made them vulnerable to other targets (Gremke et al., 2020). Cancer cells resistant to platinum compounds demonstrated an acquired resistance through increased MTORC1 protein complex. Increases in MTORC1 lead to the suppression of autophagy and created vulnerabilities to metabolic inhibitors (2DG, DCA, metformin, phenformin, AZD7457) (Gremke et al., 2020). This vulnerability should be exploited in the clinical setting to achieve synthetic lethality in the future.

Perhaps one of the clearest indicators of autophagy’s relationship to cell death is seen through multiple studies performed with gene knockdown/knockout of autophagy related proteins. In these studies, genetic ablation of autophagy genes has been seen to decrease cell death pathways including LMP-associated death (the result of lysosomal contents being released into the cell due to lysosomal permeabilization), Pyroptosis (inflammatory dependant cell death due to cytokine release *via* inflammasome activation), and Ferroptosis (iron dependant cell death that is instigated by lipid peroxidation). These studies are summarised in Table 3.

**TABLE 3:** The effect of autophagy gene alteration on cell death pathways.

Gene/protein	Gene alteration	Model	Type of cell death	Modulation that occurred	Reference
ATG13	KO	*In Vitro* MEFs	Ferroptosis	Decreased	Gao et al. (2016)
ATG3	KO	*In Vitro* MEFs	Ferroptosis	Decreased	Gao et al. (2016)
ULK1/2	KO	*In Vitro* MEFs	Ferroptosis	Decreased	Gao et al. (2016)
ATG5	KO, KD	*In Vitro* Cell lines: MEFs, PANC1, PANC2.03, HT-1080, Y79-CR, HepaG2	Ferroptosis	Decreased	Gao et al. (2016), Hou et al. (2016), Bai et al. (2019), Liu et al. (2022)
		*In Vivo* Model: HepaG2 cells subcutaneously injected in 6- to 8-week-old athymic nude or B6 mice			
ATG7	KO, KD	*In Vitro* Cell lines: MEFs, PANC1, PANC2.03, HT-1080	Ferroptosis	Decreased	Hou et al. (2016)
		*In Vitro* Cell line: C17.2	LMP-Associated cell death	Decreased	Walls et al. (2010)
		*In Vivo* ER-Cre: *atg7* ^*fl/fl*^mice	Pyroptosis	Increased	Pu et al. (2017)
ATG16L1	KO	*In Vitro* Cell line: MEFs	Pyroptosis	No change	Saitoh et al. (2008)
		*In Vivo* Atg16L1-deficient C57BL/6 mice			
ATG12	KD	*In Vitro* Cell line: Hek293	Apoptosis	Decreased	Rubinstein et al. (2011)
BECN1	OE	*In Vitro* Cell lines: HCT116, CX-1, HT1080	Ferroptosis	Increased	Song et al. (2018)
	KD	*In Vitro* Cell lines: HCT116, CX-1	Ferroptosis	Decreased	Song et al. (2018)
SQSTM1	KD	*In Vitro* Cell line: MEFs	Ferroptosis	Decreased	Yang et al. (2019)

KO, knock out; KD, Knock Down and OE, over expression.

##### Potential cancer adaption to autophagy inhibition

Inhibition of autophagy for prolonged periods of time, like with any drug, may lead to resistance. In fact, some research has suggested that by blocking autophagy several other non-canonical autophagy pathways and the Nrf2 pathway are upregulated to circumvent this loss.

In a study by Towers and colleagues it was determined that even autophagy dependent cell lines could adapt and survive despite the knockout of Atg7, and that the Atg7 null cell population had an upregulation of Nrf2 and the Nrf2 signalling pathway. This upregulation of Nrf2 resulted in increased proteasomes, increased cell growth, decreased apoptosis and contributed to therapeutic resistance (Towers et al., 2019). Evidence has suggested that this is due to an increase in p62 availability. Nrf2 when bound to Keap1 is inhibited, however, if Keap1 is bound to p62 instead then Nrf2 remains active (Komatsu et al., 2010; Towers et al., 2019).

Additionally, a phase I clinical trial performed on canines with lymphoma was done to evaluate the effect of combining HCQ with doxorubicin. The initial findings of this study were promising as canines demonstrated a high overall response rate of 93.3% (Barnard et al., 2014). However, progression-free interval was only observed to be around 5 months (Barnard et al., 2014). In a comparable study of canines treated with doxorubicin alone a similar progression free interval of 5.6 months was observed (Lori et al., 2010).

Thus, some cancer cells may adapt to autophagy inhibition by upregulating other stress pathways. Furthermore, cellular adaptions to autophagy inhibition like the upregulation of the Nrf2 pathway may then create a cell-fate determining vulnerability to Nrf2 targeted therapies which should be explored.

## Discussion/conclusion

Therapeutic resistance remains a major area for concern in cancer treatment and significantly impacts upon patient outcomes. Research into the specific mechanisms by which the cancer cell develops therapeutic resistance has highlighted the cellular process known as autophagy.

Autophagy is upregulated in numerous cancers, and increasing evidence demonstrates that its inhibition, either through genetic knockdown or by pharmacological compounds, has improved the cell’s response to therapeutics. Furthermore, increasing evidence suggests that autophagy can influence therapeutic resistance due to its inverse relationship with apoptosis and its influence on DNA damage repair pathways.

PI3K and lysosomal inhibitors have demonstrated promising results in overcoming therapeutic resistance in cancer cells. However, several factors should be considered before their use in the clinical setting.

On a broader scale, autophagy is essential for maintaining cellular homeostasis in healthy cells. The wide-scale targeting of autophagy may prevent its cytoprotective roles and lead to the toxic buildup of damaged and out-lived proteins and organelles in otherwise healthy tissue. The ability of autophagy to degrade large out-lived, and toxic materials is one of the reasons why its decline is hypothesised to be involved in several pathologies, including ageing and neurodegenerative diseases like Huntington’s and Parkinson’s (McAfee et al., 2012; Akin et al., 2014; Bosc et al., 2018; Xu et al., 2018; Fu et al., 2019). Thus, the effect of long-term treatment on the organs of the nervous system should be considered in any treatment regime.

More specifically, several factors about the current PI3K and lysosomal inhibitors should be considered before future use. Firstly, the specificity of the PI3K inhibitors has not been consistent in targeting class III PI3K until recently. Although more specific derivatives have been developed, most of the research done to date has been with older inhibitors. In addition to this, autophagy can be activated *via* non-canonical pathways. Thus, targeting PI3K may not have the capacity to completely inhibit autophagic activity as the cells could potentially circumvent the initiation complex and still have upregulated autophagy in the presence of PI3K inhibitors. As both canonical and non-canonical autophagy pathways require the lysosome, targeting the lysosome may prove a more beneficial and effective approach.

The benefit of targeting the lysosome extends past it being just a more specific target but also introduces the inhibition of, not only macroautophagy, but also the alternative autophagic pathways of chaperone-mediated autophagy and micro autophagy. In contrast to the benefits, a potential disadvantage of targeting the lysosome is that emerging evidence shows that lysosomes have independent roles and are not just specific to autophagy. Furthermore, lysosomes are fused with macrophages and dendritic cells and aid with the clearance of foreign microbes and antigen presentation (Xu et al., 2018). Thus, the immune system may be adversely affected by targeting the lysosomes indiscriminately.

Of interest is an apparent trend in the glycolytic phenotype being the most sensitive or responsive to autophagic inhibition. It has been presented as a consistent predictor in blood cancers (Chen et al., 2014b) and solid tumours (Cohen et al., 2022). However, it is suggested efficacy would be increased as an adjuvant with some of the frontline chemotherapeutics that result in a shift in metabolic programming as a means of evading their effects, such as Cisplatin (Xu et al., 2022), 5FU (Denise et al., 2015) and Doxorubicin (McGuirk et al., 2021).

The current autophagy inhibitors have several potential drawbacks, as discussed above. Research into autophagic mechanisms and the differences between physiological autophagy and pathologic autophagy is warranted. Delineating molecules specific to autophagy in the context of cancer would also be beneficial as it highlights potential therapeutic targets. Several macro autophagy-specific molecules could also be targeted in the future, including the Atg’s and LC3 involved in autophagosome formation. Targeting these molecules may reduce off-target effects like the immune system. Theoretically, the alternative to suppressing autophagy would be to stimulate and cause an over-activation of the process to bring about dysfunction, particularly in the context of cancer cell but potentially positively regulating the immune system (Yonekawa and Thorburn, 2013; Karsli-Uzunbas et al., 2014).

Despite these considerations, autophagy inhibitors are a very promising co-treatment for aggressive and resistant cancers whose advantages potentially outweigh their disadvantages. Further investigation into current autophagy inhibitors in therapeutic resistance is warranted, especially in the context of cancers like pancreatic cancer and GBM, whose survival rate is abysmal due to resistance to current treatments. Furthermore, a sustained effort should be made to research and understand the mechanism of the “pathologic” form of autophagy so that more specific and directed autophagy inhibitors can be developed and used to treat advanced and therapeutically resistant cancers more precisely.

## Figures and Tables

**Table:** 

**3-MA**	3-methyladenine
**4EBP1**	4E-binding protein 1
**Akt**	Protein kinase B
**ALK**	Anaplastic lymphoma kinase
**AML**	Acute myeloid leukaemia
**AMP**	Adenosine monophosphate
**AMPK**	AMP-activated protein Kinase
**ATG/Atg**	Autophagy related proteins
**ATM**	Ataxia-telangiectasia mutated
**ATP**	Adenosine triphosphate
**BAD**	BCL2 associated agonist of cell death
**BafA1**	Bafilomycin A1
**BAK**	Bcl-2 homologous antagonist/killer
**BAX**	Bcl-2-associated X protein
**Bcl-2**	B-cell lymphoma 2
**Bcl-XL**	B-cell lymphoma-extra large
**BID**	BH3 interacting-domain death agonist
**BIK**	Bcl-2-interacting killer
**CMA**	Chaperone Mediated autophagy
**CML**	Chronic myeloid leukaemia
**CQ**	Chloroquine
**DDR**	DNA damage response
**DNA**	Deoxyribonucleic acid
**ER**	Endoplasmic reticulum
**ERGIC**	Endoplasmic reticulum-Golgi intermediate compartment
**EIF**	Eukaryotic initiation factor
**GBM**	Glioblastoma
**GTP**	Guanosine triphosphate
**GβL**	mammalian lethal with sec-13
**HCC**	Hepatocellular carcinoma
**HCQ**	Hydroxychloroquine
**HOPs**	homotypic fusion and vacuole protein sorting
**IC**	Inhibitory concentration
**IGF**	Insulin-like growth factor
**IL-6**	Interleukin 6
**Keap1**	Kelch-like ECH-associated protein 1
**LC3**	Microtubule-associated protein light chain 3
**LMP**	Lysosomal Membrane Permeabilization
**Mcl-1**	myeloid cell leukemia sequence 1
**mTOR**	Mammalian target of rapamycin
**Nrf2**	nuclear factor erythroid 2–related factor 2
**NSCLC**	Non-small cell lung cancer
**PI3K**	phosphoinositide 3-kinase
**PI3P**	Phosphatidylinositol 3-phosphate
**PLEKHM1**	Pleckstrin Homology and RUN Domain Containing M1
**PRAS40**	Proline-rich Akt substrate of 40 kDa
**PTEN**	Phosphatase and tensin homolog
**RAPTOR**	regulatory-associated protein of mTOR
**RILP**	Rab-interacting lysosomal protein
**RITA**	Reactivating p53 and Inducing Tumor Apoptosis
**ROS**	Reactive oxygen species
**SK61**	S6 protein kinase 1
**SAHA**	Suberoylanilide hydroxamic acid
**Sal**	Salidroside
**SNAP-29**	synaptosome associated protein 29
**SNARE**	Soluble N-ethylmaleimide-sensitive factor activating protein receptor
**STX17**	Syntaxin 17
**TGA**	Therapeutic goods administration
**TMZ**	Temozolomide
**TORC1**	mammalian target of rapamycin complex 1
**TSC2**	Tuberous sclerosis 2
**ULK**	Unc-51 Like Autophagy Activating Kinase 1
**UVRAG**	UV radiation resistance-associated gene
**VAMP**	Vesicle-associated membrane protein
**V-ATPase**	Vacuolar ATPase
**VPS**	Vacuolar protein sorting
**WIPI**	WD-repeat protein interacting with phosphoinositides
**γH2Ax**	Phosphorylated form of the H2A histone family member X
